# Geographical and Seasonal Analysis of the Honeybee Microbiome

**DOI:** 10.1007/s00248-022-01986-x

**Published:** 2022-03-14

**Authors:** Eduardo L. Almeida, Celine Ribiere, Werner Frei, Denis Kenny, Mary F. Coffey, Paul W. O’Toole

**Affiliations:** 1 School of Microbiology and APC Microbiome Ireland, University College Cork, Cork, T12 K8AF Ireland; 2 Keeling’s Farm, Food Central, St. Margaret’s, Co. Dublin, K67 YC83 Ireland; 3 Department of Agriculture Food & the Marine, Backweston Campus, Celbridge, Co. Kildare, W23 X3PH Ireland

**Keywords:** Honeybee, *Apis mellifera*, Microbiota, Microbiome

## Abstract

We previously showed that colonies of thriving and non-thriving honeybees co-located in a single geographically isolated apiary harboured strikingly different microbiomes when sampled at a single time point in the honey season. Here, we profiled the microbiome in returning forager bees from 10 to 12 hives in each of 6 apiaries across the southern half of Ireland, at early, middle, and late time points in the 2019 honey production season. Despite the wide range of geographical locations and forage available, apiary site was not the strongest determinant of the honeybee microbiome. However, there was clear clustering of the honeybee microbiome by time point across all apiaries, independent of which apiary was sampled. The clustering of microbiome by time was weaker although still significant in three of the apiaries, which may be connected to their geographic location and other external factors. The potential forage effect was strongest at the second timepoint (June–July) when the apiaries also displayed greatest difference in microbiome diversity. We identified bacteria in the forager bee microbiome that correlated with hive health as measured by counts of larvae, bees, and honey production. These findings support the hypothesis that the global honeybee microbiome and its constituent species support thriving hives.

**Supplementary Information:**

The online version contains supplementary material available at 10.1007/s00248-022-01986-x.

## Introduction

The honeybee *Apis mellifera* occupies an unusual position in conservation biology: it is a species that faces many threats despite its artificial introduction into all continents except Antarctica; it is a species considered to be domesticated livestock by many conservationists; it survives existential threats in some regions largely due to human protective measures; it is promoted at the expense of native bees in some locations; it is indispensable for human food production and is bred and managed on an industrial scale to facilitate the mass production of nuts and fruit that are dependent on it for pollination [1, 2]. In many respects, *Apis mellifera* is therefore an emblematic species for the complex global ecological and sustainability challenges we face.

Colony collapse disorder (CCD) has previously caused catastrophic losses to hobby apiaries, honey producers and to commercial pollination operations, particularly in North America. Though the precise reasons for CCD are still under investigation, infections by the parasite *Varroa destructor* that increase the susceptibility of bees to viral pathogens is emerging as a primary factor [3, 4]. This may be exacerbated by the usage of a group of pesticides known as neonicotinoids that have been widely applied to crops, horticulture and pets, and that interfere with honeybee navigation, circadian rhythms and sleep [5–7]. A ban on outdoor application of three neonicotinoids (clothianidin, imidacloprid and thiamethoxam) was implemented by the European Union in 2018. Apart from these factors, honeybees are susceptible to infectious diseases caused by bacterial, fungal, viral and microsporidial pathogens [8] that can cause significant losses to apiaries, even greater than CCD in many geographical regions, and that must be managed by a combination of surveillance, prompt treatment, or in some cases (e.g. foul-brood), selected colony destruction.

Thriving bee hives are obviously desirable because they produce more forager bees, more efficient pollination, greater honey yields, and greater potential for increasing hive numbers through splits and queen-generation, but they also have greater resistance to common chronic endemic diseases such as nosemosis [9] and chalkbrood [10]. Protein nutrition achieved through pollen gathering is an important determinant of resistance to microbial pathogens [11]. In recent years, interest has grown in the role of the honeybee microbiome in modulating health and disease risk [12–14]. Multiple studies have shown that the microbiome is dominated by 5–8 bacterial phylotypes [15, 16] that are involved in digesting the main dietary ingredients, nectar (carbohydrate) and pollen (protein) [17, 18]. There are also indications of microbiome-pathogen susceptibility interactions, since the intensity of *Nosema ceranae* infection is related not to global microbiome composition but to the relative abundance of a few key taxa such as *Gilliamella* spp. [19].

We previously studied the microbiome in a single apiary in an isolated peninsula in south-western Ireland and reported that variation in microbiota composition and relative abundance exist between workers within the same colony and in between hives [20]. We also noted differences in the abundance of taxa associated with carbohydrate and protein degradation, which were higher in thriving and non-thriving hives respectively [20]. We suggested these differentially abundant taxa might be useful as biomarkers or intervention points for promoting hive health. An obvious limitation of that study was its cross-sectional nature and single location. Here, we studied 6 apiaries across southern Ireland, surrounded by different forage types, and surveyed them across the 2019 honey production season, to investigate diet-microbiome-health interactions in honeybees and more specifically the influence of location and time point in the honeybee microbiome.

## Materials and Methods

### Specimen Collection

**Fig. 1:**
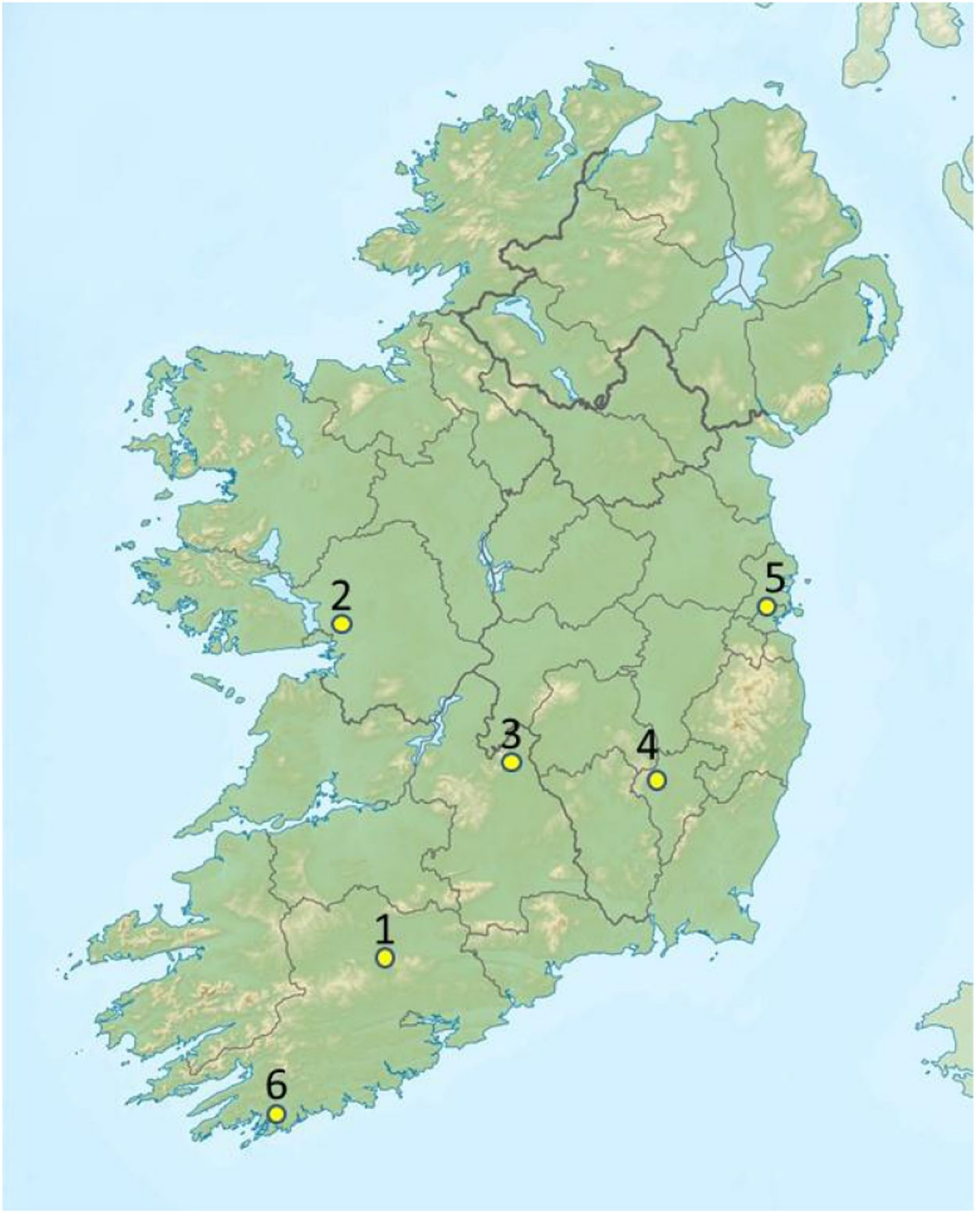
Location of the 6 apiaries where honeybee microbiome profiling was performed throughout the 2019 season. To maintain apiary/beekeeper confidentiality, locations are approximate only. Map adapted from: Wikimedia Commons, Author: Nilfanion, under the Creative Commons Attribution-Share Alike 3.0 Unported license

Bee samples were collected from 6 apiaries in southern Ireland at map locations shown in Fig. 1. Sampling dates are provided in Supplementary Table 1. All apiaries used National, Commercial or Rose hives. All hives were wooden except Apiary 5, which used Swienty Styrofoam hives. Apiaries were sampled at 3 time points throughout the 2019 season, spanning from April 28th to September 7th. Apiary 1 (North County Cork) was additionally sampled to collect debris from the hive floor/varroa insert board, from which the microbiome of the physical hive environment was measured.

For collecting bees, 10 to 12 hives were selected per apiary and serially sampled on the indicated dates. Bees were collected when it was not raining and when the temperature was above 12 °C—conditions when forager bees are typically airborne in Ireland. The hive entrances were blocked with foam rubber and returning foragers were collected in sterile 50-ml Falcon tubes at the hive entrance. The majority but not necessarily all bees had pollen in their corbiculae. The bees were euthanized by rapid cooling on ice, then transported same day under refrigeration to the laboratory where they were stored at − 80 °C until DNA was extracted.

To analyse hive debris samples, material was recovered into a sterile collection tube from the Varroa insert board under the open-mesh floor of the National brood boxes from Apiary 1. This material consisted of wax cap fragments, bee body detritus, occasional moths and other debris.

A sample of a commercial bee fondant feed (Candipolline, Enolapi SRL, Italy) was collected from Apiary 2.

### DNA Extraction and Bacterial 16S rRNA Gene Amplicon Sequencing

Total bee DNA was extracted from 908 whole bees (forager) samples (6 apiaries, 3 time points, 10 or 12 hives, 4 or 5 bees each) plus 36 hive debris samples (1 apiary, 3 time points, 12 hives) using the Qiagen POWER SOIL pro kit according to the manufacturer's instructions. The hive debris material and the commercial bee fondant samples were suspended in sterile saline solution, bacteria were released by vigorous vortexing, and the soluble phase was recovered for DNA extraction. To analyse the total bee microbiome, libraries for 16S rRNA gene sequencing were prepared using Phusion HF DNA polymerase and V3/V4 specific primers including the Illumina adapter sequence and unique 8 nt dual indices (Illumina Nextera XT indices) as previously described by our laboratory [20]. Samples were DNA sequenced over 5 runs on the Illumina MiSeq Platform (600 cycles per run, paired end, 2 × 300 bp, approx. 44 million DNA sequence reads) by Teagasc, Moorepark, Co. Cork.

### Bioinformatics and Biostatistical Analyses

Raw reads were processed for quality filtering and trimming using DADA2 (version 1.18) (parameters trimLeft = 15, truncLen = 240, maxEE = 2) in R (version 4.0.3) [21, 22]. Only forward reads were further processed and analysed due to decreased quality of the reverse reads, which can negatively affect sample inference in the DADA2 pipeline. Reads dereplication, learning of the error rates with randomised reads, and sample sequence variant inference with pooled samples were performed using DADA2. Construction of amplicon sequence variant (ASV) table and removal of chimeras were performed using DADA2, followed by taxonomy assignment and species assignment using DADA2 and the SILVA v138.1 database [23]. Rare ASVs were removed, keeping only those present in at least 10% of the samples per apiary and sample type. All microbiome composition analyses were performed at ASV level, unless specified otherwise.

At each apiary inspection, we recorded the number of frames (FR) of bees, brood, pollen and honey in each hive when available (*n* = 182, frames of bees *n* = 172, brood *n* = 180, honey *n* = 154, pollen *n* = 68; Supplementary Table 2 and Supplementary Fig. 1). We refer to these as the hive metadata. As a generalization, the higher these numbers, the healthier the hive. Spearman correlations of the mean relative abundance of individual bacterial taxa in the microbiome of hives with their respective metadata values were computed and represented as heatmaps.

All biostatistical and microbial community analyses were performed in R and RStudio (version 1.5.46) [24], with the packages phyloseq (version 1.36) [25], vegan (version 2.5–7) [26], ade4 (version 1.7–16) [27], ggpubr (version 0.3.0) [28], psych (version 2.1.3) [29] and dunn.test (version 1.3.5) [30]. Graphics were generated in R using the packages ggplot2 (version 3.3.3) [31], ComplexHeatmap (version 2.8.0) [32] and ggpubr. Unless specified otherwise, statistical significances were determined employing the non-parametric Kruskal–Wallis’ test and the Dunn’s post hoc test, or Wilcoxon’s test when specified, with *p* values < 0.05 considered significant, all of which were adjusted for false discovery rate (FDR) using the Benjamini–Hochberg method.

### Sequence Data Accession Number

All 16S rRNA gene sequence data are available through the European Nucleotide Archive (ENA) database under the accession number PRJEB47333.

## Results

### The Honeybee Microbiome Is Distinct from That of Hive debris

For this longitudinal microbiome survey, we chose 6 apiaries covering southern Ireland (Fig. 1) and a variety of location types and forage regimes (Table 1). Although most of the apiaries were close to farmland, only half had other apiaries within a 5 km flying radius. Apiary 4 was on an agricultural research station surrounded by oil seed rape fields, which was associated with bees that reacted aggressively during sampling. The colonies in Apiary 5 were located on the premises of a commercial fruit grower with large areas of outdoor fruit, and fruit under glass (with open windows) supplied with commercial bumblebee pollinators. Apiary 6 was located in a remote coastal setting, but was not the same apiary surveyed in our previous study [20].

**Table 1:** Locations and properties of the apiaries subjected to microbiome analysis in this study

Apiary	Location	Apiary setting
1	North Co. Cork	Surrounded by tillage and dairy; southerly aspect; other apiaries within 5 km
2	Central Co. Galway	Surrounded by sheep and cattle grazing; other apiaries within 5 km
3	North Co. Tipperary	Grassland for cattle raising
4	North Co. Carlow	Central plains; close to oil seed rape crop and government agricultural/ horticultural station; other apiaries within 5 km
5	North Co. Dublin	Managed pollinator apiary on lands of large commercial fruit grower with indoor and outdoor horticulture
6	West Co. Cork	Remote coastal apiary bordered by mixed tillage and pastoral land

**Fig. 2:**
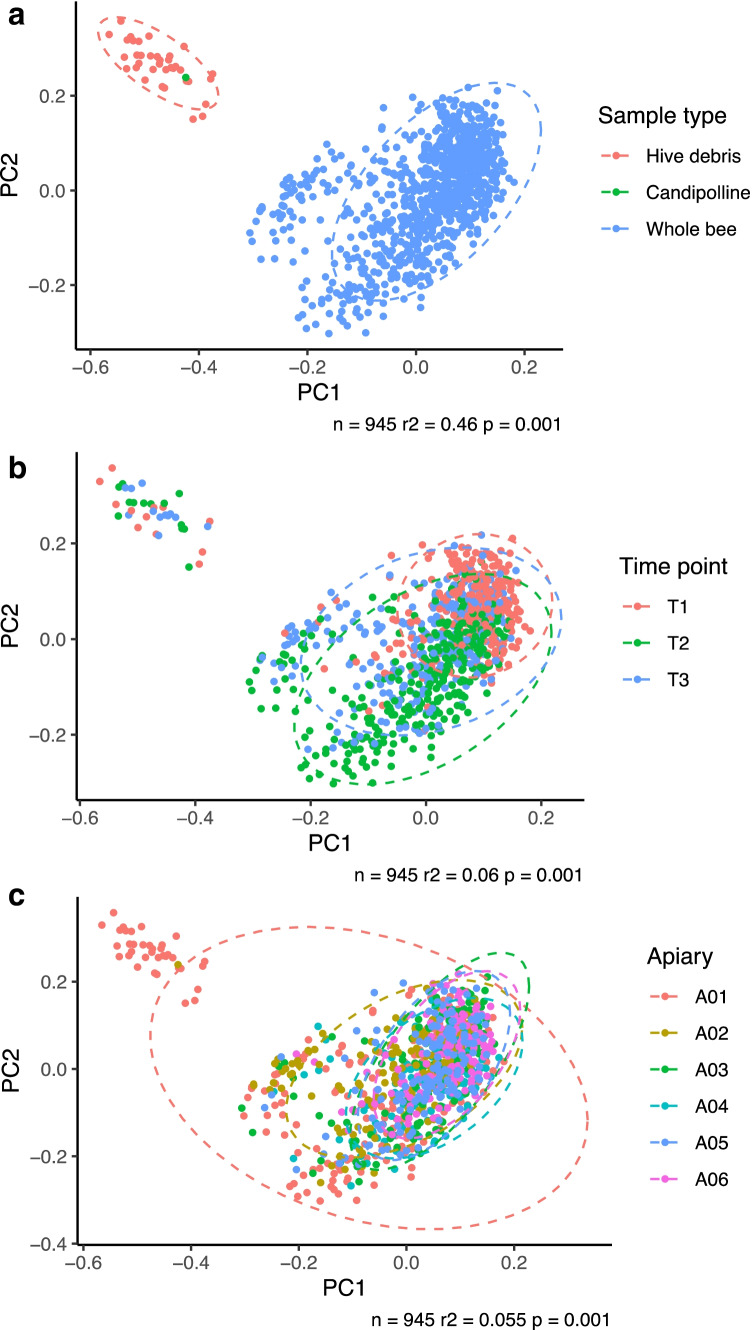
Principal co-ordinate analysis of the honeybee microbiome across all apiaries, all time points

Based upon our previous study [20], where we established the whole-body microbiota as reliably representing the gut microbiome while also capturing the whole-body microbial exposure that was expected to vary during the season, we similarly extracted total bacterial DNA from both the hive debris samples and a representative fondant sample, profiled the microbiome by 16S rRNA gene amplicon sequencing and compared the microbiome to that of all the bees sampled. Principal co-ordinate analysis (PCoA) of microbiome profiles was performed at amplicon sequence variant (ASV) level which gives maximal discrimination, revealing that the data clustered according to sample type (i.e. whole body from foragers (WB) or hive debris (HD); Fig. 2A). The microbiome present in the commercial fondant was also distinct from that of the whole-bee samples, while more similar to the hive debris samples (Fig. 2A). The honeybee microbiome was distinct from that of the hive debris, collected from Apiary 1 at a single timepoint, which confirms that the microbiome data collected are derived from the bee rather than the physical hive environment, or supplementary feeding at the first time point.

### The Honeybee Microbiome Does not Cluster by Apiary but Clusters by Time Point

**Fig. 3:**
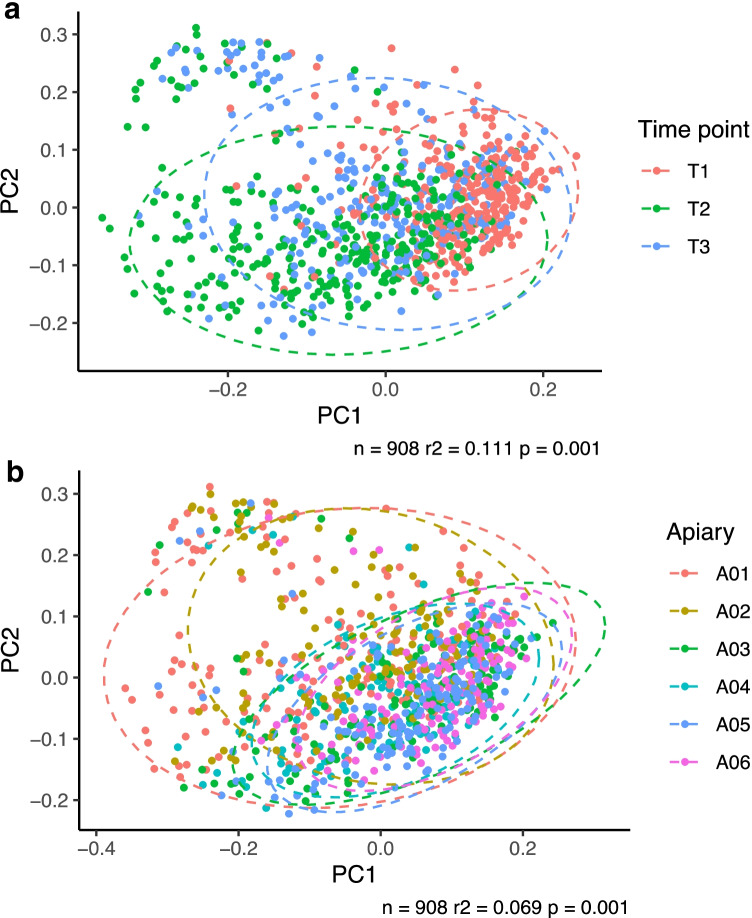
Principal co-ordinate analysis of the honeybee microbiome across all apiaries, all time points, whole-bee microbiome of foragers only (i.e. hive debris and fondant samples excluded)

A hypothesis at the beginning of this project was that the physical location of an apiary and the locally available forage (as shown in Table 1) would have a strong influence on the honeybee microbiome, over-riding other factors. However, when we excluded the hive debris and fondant samples and performed β-diversity analysis (all whole-bee samples, all time points), there were clear clustering and separation of the honeybee microbiome by time point, independent of apiary identity (Fig. 3A). In contrast, there was no clear separation of the bee microbiome by apiary (Fig. 3B), and thus, the location/geography/local forage did not appear to be a major determining factor for the bee microbiome. Furthermore, although statistical (envfit) analyses identified significant correlation between plot ordinations for both time point and apiary groups, the squared correlation coefficient (*r*^2^) was higher for time point than for apiary (*r*^2^ = 0.111 and 0.069, respectively), as shown in Fig. 3A and B, respectively. Additionally, a visible difference in the dispersion by apiary could be observed in the PCoA plot, which was further confirmed using the betadisper function (*p* = 0.001), with pairwise comparisons showing that Apiaries 1 and 2 had similar levels of dispersion (*p* = 0.96), while being different from all other apiaries (*p* < 0.05). This observed difference in dispersions seemed to be particularly caused by samples clustering at the upper section of the ordination plot (Fig. 3A), and Spearman correlations (*r* >|0.4| and *p* adjusted < 0.05 cutoffs) between the PC2 axis and ASV relative abundance indicated that this was driven to some extent by an increased relative abundance of *Arsenophonus* ASVs, for which 8 ASVs were positively correlated with PC2. Conversely, negative correlations were detected with the relative abundance of ASVs classified as *Bartonella apis*; *Bifidobacterium* (which also had 2 ASVs positively associated with PC2), *Bifidobacterium indicum*, *Candidatus Profftella*, *Frischella*, *Frischella perrara*, *Gilliamella*, *Gilliamella apicola*, *Orbaceae*, *Rhizobiaceae* and *Snodgrassella*.

The tightest clustering of the bee microbiome data was early in the season (time point 1 or T1). Meanwhile, at time point 2 (T2), the microbiome had shifted to a more dissimilar composition across all apiaries/hives, with the exception of Apiary 5 (Supplementary Fig. 2). This time point coincided with either at the end of the “June gap” in nectar flow in Ireland (which is quite regional and local in timing), or in early summer flow in most apiaries. In the last time point (T3), which was after honey harvest, the microbiome had moved back to being more similar to the starting microbiome, suggesting loss of the effect of the summer forage—again, with the exception of Apiary 5, which appeared to have a more continuous change in microbiota composition over time points (Supplementary Fig. 2). The extent to which samples clustered by time point varied; this was visible from examining the beta diversity separately for individual hives in individual apiaries (Supplementary Fig. 2). Specifically, weaker correlations between microbiome composition and time point were detected in hives from Apiaries 2, 4, and 6.

### The Honeybee Microbiome Alpha Diversity and Composition Changes During the Season

**Fig. 4:**
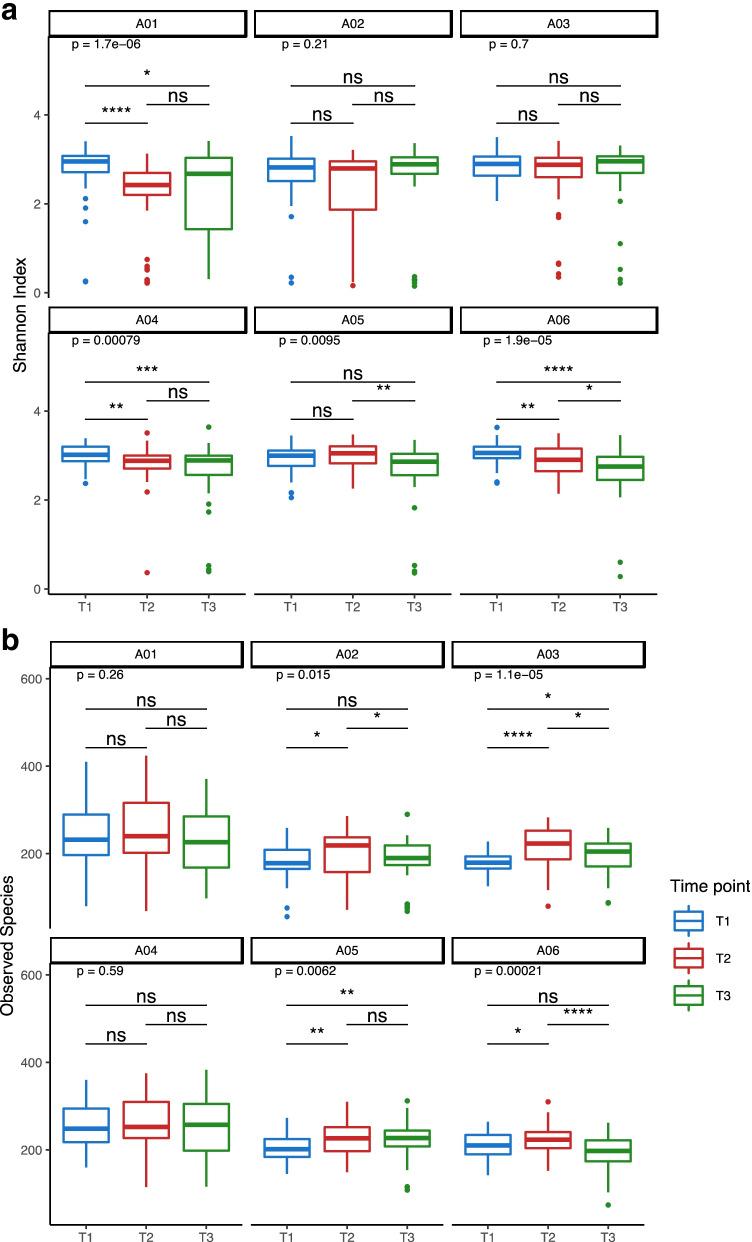
Alpha diversity in whole-bee microbiome over time by apiary as measured by **A** Shannon index; **B** observed species. Pair-wise comparisons that reach statistical significance are asterisked; ns = not significant

The diversity of an ecosystem is—in most circumstances—a good indicator of ecosystem health. This is a generalization, and it depends on the nature of the ecosystem, and what index of alpha diversity is used. In humans for example, loss of alpha diversity in the gut microbiome may be an indicator of risk or state of disease [33]. For the microbiome, one can measure the number of species, their richness, or evenness. We measured Shannon, Simpson, and Chao1 indices and Observed Species (Fig. 4, Supplementary Fig. 3), ultimately focusing on the Shannon index as it accounts for both abundance and evenness of the species present [34].

Within individual apiaries, we detected the greatest variation and range in the Shannon index at the second time point, which also corresponded to the time when the microbiome had become most different from the beginning and end of the season (Fig. 4). Apiary 1 displayed a high level of variability of alpha diversity at the third time point, which may have been related to recent i.e. late honey harvest, thus later than usual nectar collection. Measurement of the changes in alpha diversity over time within individual hives (Supplementary Fig. 4) revealed that hives 2, 4, 8, and 11 were largely responsible for the low alpha-diversity measurement for Apiary 1 at T3. Apiary 2 had four hives with low alpha diversity at the second time point. Apiaries 5 and 6 had significantly lower alpha diversity at the final time point than the first or second timepoints (Fig. 4), but the absolute range of values over time were relatively small.

### Correlations Between Honeybee Microbiome and Hive Health

**Fig. 5:**
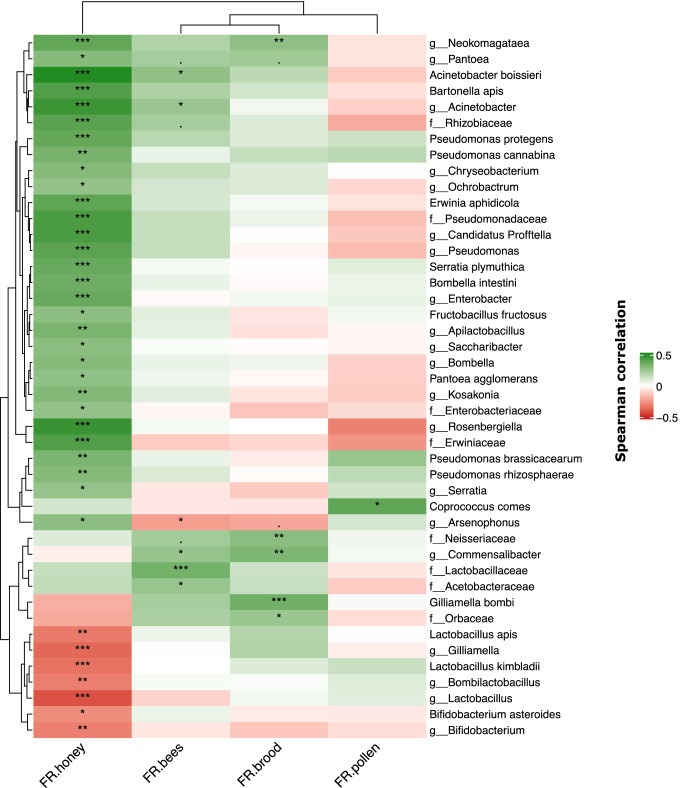
Heat-map of Spearman correlations of the abundance of bacterial taxa (agglomerated taxonomy) and metadata variables (FR honey, FR bees, FR brood, FR pollen). All WB samples were used for the analysis; Spearman correlations with FDR-corrected *p* values

The hive metadata including the number of frames (FR) of bees, brood, pollen and honey were utilised as proxies for hive health and these data were tested for correlation with differences in the relative abundance of bacterial taxa. When all time points were aggregated across all hives/apiaries for which data were collected, and all ASVs were agglomerated at their most specific taxonomic rank (Fig. 5), we identified several bacterial taxa with statistically significant correlations, either positive or negative, with the number of frames of honey, bees and brood, and only one statistically significant correlation with the number of frames of pollen. We detected statistically significant negative correlations between seven bacterial taxa and honey production.

Correlations between bacterial taxa and frames of bees and brood showed similar patterns of associations, with these two variables being more closely related in the Spearman distances dendrogram in Fig. 5. Agglomerated ASVs classified as *Commensalibacter* possessed statistically significant positive correlations with both these metadata values, while other taxa also showed this trend, although not passing the strict FDR-adjusted *p* values of 0.05 employed in this analysis. These included ASVs classified in the genus *Arsenophonus*, which were negatively correlated with frames of bees (*p* adjusted < 0.05) and frames of brood (*p* adjusted < 0.10), while positively correlated with the frames of honey (*p* adjusted < 0.05). *Coprococcus comes* was the only bacterial taxon with statistically significant association with number of frames of pollen, with a positive association and no significant associations with the other metadata values. This could have been influenced to some degree by the lower number of data points available for this parameter (*n* = 68).

When analysing correlations in finer detail at ASV-level, i.e. non-agglomerated at higher taxonomy ranks, a much larger number of statistically significant correlations were detected, including additional correlations with number of frames of pollen, although maintaining the general trend observed in the correlation analysis with agglomerated taxa (Supplementary Fig. 5 and Supplementary Fig. 6). These included a large number of ASVs assigned to the genus *Commensalibacter*, which showed strong positive associations with both number of frames of brood and number of frames of bees (Supplementary Fig. 6). This analysis also highlighted a group of bacteria whose abundance negatively associated with pollen counts, but positively associated with other metadata types, which included ASVs assigned to the families *Orbaceae* and *Rhizobiaceae*. This trend was further emphasised by determining correlations within the hive metadata values (Supplementary Fig. 7), which clearly showed strong positive correlations with number of frames of bees and number of frames of brood, as well as number of frames of honey, while these have weaker correlations with frames of pollen.

### Differentially Abundant Bacterial Taxa During the Honey Production Season

**Fig. 6:**
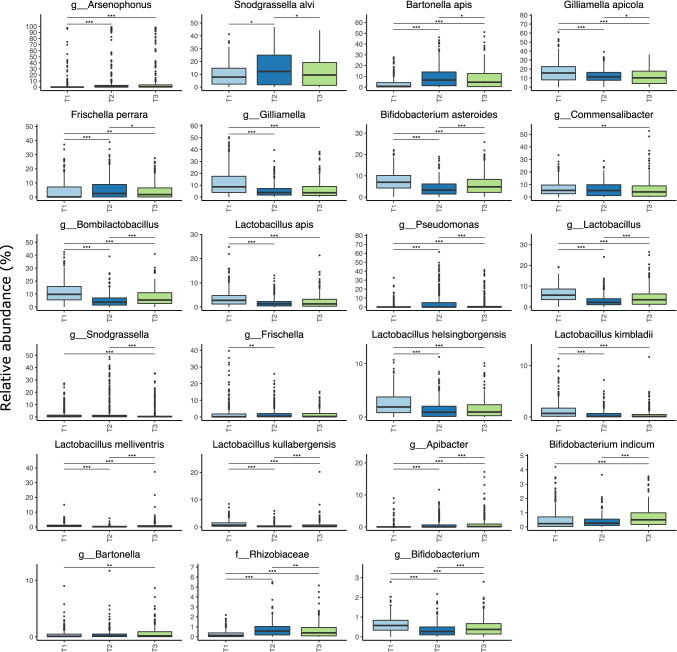
Box plots with the differentially represented bacterial taxa over time in whole-bee samples in all apiaries/hives (agglomerated at most specific taxa rank). Only taxa with at least 1% relative abundance in at least 10% of the samples have been considered for analyses. Statistically significant differences were computed with Kruskal–Wallis test and Dunn test, with a FDR-adjusted *p* < 0.05 considered significant

We investigated if bacterial taxa were differentially represented in whole-bee samples across the different time points (Fig. 6). For this analysis, only agglomerated taxa with at least 1% relative abundance in at least 10% of the samples were retained, which resulted in a total of 23 differentially abundant taxa. Interestingly, 5 of these included ASVs assigned to *Lactobacillus* species, namely *Lactobacillus apis*, *L. helsingborgensis*, *L. kimbladii*, and *L. melliventris* and *L. kullabergensis*, all of which were more abundant in T1. In contrast, although the relative abundance of ASVs belonging to the genus *Arsenophonus* varied considerably between whole-body samples, these were less abundant at T1 when compared to other time points. Conversely, ASVs belonging to the genus *Gilliamella*, including *Gilliamella apicola*, were higher in T1. These observations are in keeping with the overall microbiome compositions at Family and Genus levels per apiary over time points, as shown in Supplementary Fig. 8 and Supplementary Fig. 9.

To further assess a possible co-differential abundance of these agglomerated taxa, Spearman correlations between taxon relative abundance were also computed, aiming to identify taxa with positively or negatively associated prevalence over time (Supplementary Fig. 10). Indeed, this analysis showed that most taxon groups determined to be differentially abundant also had their relative abundances significantly correlated to at least one other taxon, with *Bifidobacterium indicum* and *Bifidobacterium* spp. being the only two exceptions.

## Discussion

A main objective of this project was to determine the influence of location and time point, i.e. the seasonal and forage effects, upon the bee microbiome. We thus analysed the honeybee microbiome from 6 apiaries from varied environments across southern Ireland over the 2019 honey production season, also seeking to identify associations and/or patterns between microbiota composition and hive “health” or productivity. The microbiota composition of samples derived from apiaries in very different environments was more similar to each other within time points than across time points in the same apiary, i.e. apiary location was not the strongest factor driving differences or changes in the honeybees’ microbiota. This finding is broadly in line with the observation that the microbiome of corbiculate bees is largely stable [35], and perhaps site differences would be detectable in future studies by investigating strain-level resolution of the microbiome [36]. However, achieving this level of detail would require the employment of shotgun metagenomics rather than 16S rRNA data, which would be challenging for the scale and number of samples in the present regional study, across an entire season. Correlations between microbiota composition and time points were weaker in apiaries 2, 4 and 6, when compared to the other apiaries. This could potentially be due to their respective locations or other factors playing stronger roles in microbial composition changes, considering that Apiary 2 colonies were being fed with Candipolline, while Apiary 4 is located in an agricultural research area, and Apiary 6 is located in a remote peninsular area. Also notable was the fact that samples from time points 1 and 3 were more similar among each other in most apiaries, with a stronger compositional shift observed in time point 2. This was not the case, however, for Apiary 5, whose samples became increasingly different over time (i.e. T1 is more similar to T2 rather than T3), perhaps due to a higher degree of human intervention and management in this apiary, operated as a fixed pollinator site for commercial fruit production, or as a result of a potential additional diversity in pollen supplied in the area by both commercial crops as well as several surrounding managed wildflower areas. Another hypothesis that could explain these observations regarding the influence of the time of the year on the microbiome composition is that some winter bees were sampled in T1 and T3, rather than summer bees which were more likely to be found in higher abundance in T2. Long-lived winter bees have a life expectancy of 6 months or more [37], and can persist in colonies up until May and reappear in colonies around September. Importantly, they are known to be physiologically different from summer bees in many ways, including in their gut microbiota composition [38].

We identified differences in abundances of bacterial taxa as well as changes of taxon abundance over time. Though it is hard to convey the complexity of the patterns at this level of scale, some potentially interesting signatures could be observed in the microbiome composition. For example, we noted differences in abundance of the genera *Arsenophonus* and *Lactobacillus*, the former being a known insect endosymbiont that could be related to generally poor hive health [39–41], and the latter which could be related to improved hive health [40]. Some bee microbiota were identified to be constituted almost entirely by *Arsenophonus* ASVs, which could potentially indicate very poor health of these honeybees, although this somewhat extreme relative abundance was not observed in the average abundance of samples. This hypothesis is supported by previous studies which associated *Arsenophonus* species with poor honeybee colonies health [40], and identified an abnormally high abundance of *Arsenophonus* in colonies suffering from CCD [42], as well as in bumblebees infected with the eukaryotic parasite *Apicystis bombi* [43]—although the biological mechanisms underlying these observations are still lacking. We also observed an interesting trend towards lower *Arsenophonus* relative abundance with an increased relative abundance of other taxa known to be associated with honeybees’ health, such as *Gilliamella*, *Lactobacillus*, *Bombilactobacillus* (formerly referred to as *Lactobacillus* Firm-4), *Commensalibacter* and *Bifidobacterium*, as indicated by differential bacterial taxa abundance and correlation analyses [20, 44, 45]. Among these, *Commensalibacter* are considered to be part of the core microbiome of honeybees, and have also been previously linked to greater honeybee health, for example by being detected in higher occurrence in healthy colonies in comparison to those affected by European foulbrood [46]. Furthermore, although *Gilliamella* members are biologically diverse and have been reported to have various ecological associations, their abundance patterns in the current study are in line with previous reports that proposed that members of this genus could provide health benefits to honeybees, particularly with respect to the metabolism of toxic sugars [45]. Further investigation is required in order to determine whether these observed patterns and correlations are the result of antagonistic effects of these “positive” taxa against “negative” taxa such as *Arsenophonus*, or if these are linked to more complex synergistic effects on the overall bee health.

Our results also indicated numerous correlations between hive health metadata and bacterial taxon abundance, which is consistent with a number of recent studies that have increasingly shown the importance of the microbiota for hive and bee health [20, 44, 47]. These included all of the *Lactobacillus* species that showed significant correlations, while the abundance of members of the genus *Apilactobacillus*—which include bacteria formerly classified as *Lactobacillus kunkeei*—was shown to be positively correlated with honey production. This observation may be due to the compositional nature of the 16S rRNA amplicon data, for which flower-associated bacteria such as *Apilactobacillus* species [48] are expected to appear in increased loads in the forager bees as nectar gathering is increased and consequently honey production is increased, which in turn will decrease the relative abundance of other taxa, such as *Lactobacillus*, but not necessarily decrease their absolute quantities in the microbiome. In fact, it was interesting to note that many of the taxa shown to be positively correlated with honey production include flower-associated species, e.g. *Apilactobacillus*, *Fructobacillus fructosus*, *Acinetobacter boissieri*, *Neokomagataea* [48–52]. This increased prevalence and abundance in the microbiome is potentially reflective of increased nectar gathering and, consequently, increase in honey production—although a role in promoting hive productivity cannot be excluded. The clustering of honeybee microbiome by time point also relates with the clustering of plant resources by time point as plants enter and exit flowering seasons, and the fact that honeybees appear to select the same frequently encountered plants across different habitats [53].

Associations detected here included positive correlation between ASVs belonging to the genus *Apilactobacillus* (which include species formerly classified as *Lactobacillus kunkeei*) and frames of honey, as aforementioned; *Arsenophonus* ASVs negatively correlated with the number of frames of bees; and *Commensalibacter* ASVs positively correlated with both number of frames of bees and number of frames of brood. In addition to the previously discussed *Arsenophonus* and *Commensalibacter* genera, *Apilactobacillus* species have been previously reported to provide varied health benefits to honeybees, including inhibitory activity against pathogens [54–56]. More specifically, this antibacterial activity has been recently identified in a member of this genus, viz. *Apilactobacillus kunkeei* FF30-6, via a narrow-spectrum nisin variant bacteriocin, namely Kunkecin A, with strong antimicrobial activity against *Melissococcus plutonius*—the causative agent of European foulbrood [56]. This combined evidence further highlights the importance of taxa such as *Apilactobacillus* species for the health of honeybees, not only from the metabolic aspect but also with respect to the host defence mechanisms against pathogens.

Previous studies that had assessed to varying degrees the effects of season and location on the honeybee microbiome, for the most part analysed either single or a couple of locations over single or a couple of time points [36, 57, 58]; and/or focused on microbiome differences between seasons rather than within a season [57], particularly with regard to wintering bees [38, 58–60]. Therefore, our study further enriches the current knowledge regarding the influence of time point and location over the honey production season, by analysing a large number of individual samples from six different apiary locations, deliberately chosen to encompass very different environment types. However, it is important to note some limitations due to the relative abundance/compositional nature of the 16S rRNA data employed in this study. It is possible, for example, that differences in relative abundance of a bacterial taxon are affected by the increase/decrease of other taxa—such as flower- or nectar-associated—but not necessarily reflective of differences in its absolute abundance. Additionally, it is also feasible that the observed correlations reflect an increase in collected nectar, which would consequently increase the abundance of flower-associated bacterial species, such as *Apilactobacillus*. Although we have previously shown that the honeybee whole-body and gut microbiomes are generally very similar and share the same dominant core taxa [20], it is possible that seasonal- or location-dependent variations in the crop microbiome are either not detectable, or over-influential of the whole-bee microbiome, i.e. compared to what might have been detected in a comparative analysis of crop, hind gut and whole-bee microbiomes. However, with our desire to achieve statistical power from large sample numbers, dissections on that scale for so many individual bees samples, apiaries and time points were not feasible.

This is the first study investigating the microbiome composition across different locations and time points in honeybees in Ireland. In future studies, we will employ shotgun sequencing metagenomics rather than 16S amplicon sequencing, with an aim to increase bacterial species detection resolution as well as functional profiling at gene level. We may also utilise more granular honeybee health indicators assisted by electronic hive monitoring. However, our data already highlight the potential importance of the microbiota for honeybee health, a proper understanding of which might play a crucial role in the sustainable management of these important pollinators.

## Supplementary Information

Below is the link to the electronic supplementary material.

Supplementary file1 (PDF 902 KB)

Supplementary file2 (XLSX 18 KB)

## Data Availability

The nucleotide sequence data reported are available in the ENA database under the accession number PRJEB47333.

## Code Availability

Non applicable.

## References

[1] Saunders ME, Smith TJ, Rader R (2018). Bee conservation: Key role of managed bees. Science.

[2] Geldmann J, González-Varo JP (2018). Conserving honey bees does not help wildlife. Science.

[3] Budge GE, Simcock NK, Holder PJ (2020). Chronic bee paralysis as a serious emerging threat to honey bees. Nat Commun.

[4] Doublet V, Labarussias M, de Miranda JR (2015). Bees under stress: sublethal doses of a neonicotinoid pesticide and pathogens interact to elevate honey bee mortality across the life cycle. Environ Microbiol.

[5] Alburaki M, Boutin S, Mercier P-L (2015). Neonicotinoid-coated Zea mays seeds indirectly affect honeybee performance and pathogen susceptibility in field trials. PLoS One.

[6] Fischer J, Müller T, Spatz A-K (2014). Neonicotinoids interfere with specific components of navigation in honeybees. PLoS One.

[7] Tackenberg MC, Giannoni-Guzmán MA, Sanchez-Perez E (2020). Neonicotinoids disrupt circadian rhythms and sleep in honey bees. Sci Rep.

[8] Dolezal AG, Toth AL (2018). Feedbacks between nutrition and disease in honey bee health. Curr Opin Insect Sci.

[9] Holt HL, Grozinger CM (2016). Approaches and challenges to managing *Nosema* (Microspora: Nosematidae) parasites in honey bee (Hymenoptera: Apidae) colonies. J Econ Entomol.

[10] Gilliam M, Taber S, Lorenz BJ, Prest DB (1988). Factors affecting development of chalkbrood disease in colonies of honey bees, *Apis mellifera*, fed pollen contaminated with *Ascosphaera apis*. J Invertebr Pathol.

[11] Tritschler M, Vollmann JJ, Yañez O (2017). Protein nutrition governs within-host race of honey bee pathogens. Sci Rep.

[12] Raymann K, Moran NA (2018). The role of the gut microbiome in health and disease of adult honey bee workers. Curr Opin Insect Sci.

[13] Kwong WK, Mancenido AL, Moran NA (2017). Immune system stimulation by the native gut microbiota of honey bees. R Soc Open Sci.

[14] Engel P, Kwong WK, McFrederick Q (2016). The bee microbiome: impact on bee health and model for evolution and ecology of host-microbe interactions. MBio.

[15] Engel P, Martinson VG, Moran NA (2012). Functional diversity within the simple gut microbiota of the honey bee. Proc Natl Acad Sci.

[16] Kwong WK, Moran NA (2016). Gut microbial communities of social bees. Nat Rev Microbiol.

[17] Zheng H, Perreau J, Powell JE (2019). Division of labor in honey bee gut microbiota for plant polysaccharide digestion. Proc Natl Acad Sci.

[18] Engel P, Moran NA (2013). Functional and evolutionary insights into the simple yet specific gut microbiota of the honey bee from metagenomic analysis. Gut Microbes.

[19] Rubanov A, Russell KA, Rothman JA (2019). Intensity of *Nosema ceranae* infection is associated with specific honey bee gut bacteria and weakly associated with gut microbiome structure. Sci Rep.

[20] Ribière C, Hegarty C, Stephenson H (2019). Gut and whole-body microbiota of the honey bee separate thriving and non-thriving hives. Microb Ecol.

[21] Callahan BJ, McMurdie PJ, Rosen MJ (2016). DADA2: high-resolution sample inference from Illumina amplicon data. Nat Methods.

[22] R Core Team (2021) R: A language and environment for statistical computing. R Foundation for Statistical Computing, Vienna, Austria. https://www.R-project.org/

[23] Quast C, Pruesse E, Yilmaz P (2012). The SILVA ribosomal RNA gene database project: improved data processing and web-based tools. Nucleic Acids Res.

[24] RStudio Team (2020) RStudio: Integrated development environment for R. RStudio, PBC, Boston, MA. http://www.rstudio.com/

[25] McMurdie PJ, Holmes S (2013). phyloseq: An R package for reproducible interactive analysis and graphics of microbiome census data. PLoS One.

[26] Oksanen J, Blanchet FG, Friendly M et al (2020) vegan: Community ecology package. https://CRAN.R-project.org/package=vegan

[27] Thioulouse J, Dray S, Dufour A-B (2018). Multivariate analysis of ecological data with ade4.

[28] Kassambara A (2020) ggpubr: 'ggplot2' based publication ready plots. https://CRAN.R-project.org/package=ggpubr

[29] Revelle W (2021) psych: Procedures for personality and psychological research. Northwestern University, Evanston, Illinois, USA. https://CRAN.R-project.org/package=psych

[30] Dinno A (2017) dunn.test: Dunn’s test of multiple comparisons using rank sums. https://CRAN.R-project.org/package=dunn.test

[31] Wickham H (2016). ggplot2: Elegant graphics for data analysis.

[32] Gu Z, Eils R, Schlesner M (2016). Complex heatmaps reveal patterns and correlations in multidimensional genomic data. Bioinformatics.

[33] Jeffery IB, Lynch DB, O’Toole PW (2016). Composition and temporal stability of the gut microbiota in older persons. ISME J.

[34] Kim B-R, Shin J, Guevarra RB (2017). Deciphering diversity indices for a better understanding of microbial communities. J Microbiol Biotechnol.

[35] Kwong WK, Medina LA, Koch H (2017). Dynamic microbiome evolution in social bees. Sci Adv.

[36] Moran NA, Hansen AK, Powell JE, Sabree ZL (2012). Distinctive gut microbiota of honey bees assessed using deep sampling from individual worker bees. PLoS One.

[37] Behrends (2010). Learning at old age: a study on winter bees. Front Behav Neurosci.

[38] Kešnerová L, Emery O, Troilo M (2020). Gut microbiota structure differs between honeybees in winter and summer. ISME J.

[39] Nováková E, Hypša V, Moran NA (2009). Arsenophonus, an emerging clade of intracellular symbionts with a broad host distribution. BMC Microbiol.

[40] Budge GE, Adams I, Thwaites R (2016). Identifying bacterial predictors of honey bee health. J Invertebr Pathol.

[41] Yañez O, Gauthier L, Chantawannakul P, Neumann P (2016). Endosymbiotic bacteria in honey bees: *Arsenophonus* spp. are not transmitted transovarially. FEMS Microbiol Lett.

[42] Cornman RS, Tarpy DR, Chen Y (2012). Pathogen webs in collapsing honey bee colonies. PLoS One.

[43] Parmentier A, Billiet A, Smagghe G (2018). A prokaryotic-eukaryotic relation in the fat body of *Bombus terrestris*. Environ Microbiol Rep.

[44] Alberoni D, Baffoni L, Braglia C (2021). Honeybees exposure to natural feed additives: How is the gut microbiota affected?. Microorganisms.

[45] Zheng H, Nishida A, Kwong WK et al (2016) Metabolism of toxic sugars by strains of the bee gut symbiont *Gilliamella apicola*. MBio 7. 10.1128/mBio.01326-16 10.1128/mBio.01326-16 PMC5090037 27803186

[46] Erban T, Ledvinka O, Kamler M (2017). Bacterial community associated with worker honeybees (*Apis mellifera*) affected by European foulbrood. PeerJ.

[47] Bonilla-Rosso G, Engel P (2018). Functional roles and metabolic niches in the honey bee gut microbiota. Curr Opin Microbiol.

[48] Neveling DP, Endo A, Dicks LMT (2012). Fructophilic *Lactobacillus kunkeei* and *Lactobacillus brevis* isolated from fresh flowers, bees and bee-hives. Curr Microbiol.

[49] Maeno S, Nishimura H, Tanizawa Y (2021). Unique niche-specific adaptation of fructophilic lactic acid bacteria and proposal of three *Apilactobacillus* species as novel members of the group. BMC Microbiol.

[50] Patil M, Jadhav A, Patil U (2020). Functional characterization and in vitro screening of *Fructobacillus fructosus* MCC 3996 isolated from *Butea monosperma* flower for probiotic potential. Lett Appl Microbiol.

[51] Álvarez-Pérez S, Lievens B, Jacquemyn H, Herrera CM (2013). *Acinetobacter nectaris* sp. nov. and *Acinetobacter boissieri* sp. nov., isolated from floral nectar of wild Mediterranean insect-pollinated plants. Int J Syst Evol Microbiol.

[52] Morris MM, Frixione NJ, Burkert AC (2020). Microbial abundance, composition, and function in nectar are shaped by flower visitor identity. FEMS Microbiol Ecol.

[53] Jones L, Brennan GL, Lowe A (2021). Shifts in honeybee foraging reveal historical changes in floral resources. Commun Biol.

[54] Vergalito F, Testa B, Cozzolino A (2020). Potential application of *Apilactobacillus kunkeei* for human use: Evaluation of probiotic and functional properties. Foods.

[55] Arredondo D, Castelli L, Porrini MP (2018). *Lactobacillus kunkeei* strains decreased the infection by honey bee pathogens *Paenibacillus larvae* and *Nosema ceranae*. Benef Microbes.

[56] Zendo T, Ohashi C, Maeno S (2020). Kunkecin A, a new nisin variant bacteriocin produced by the fructophilic lactic acid bacterium, *Apilactobacillus kunkeei* FF30-6 isolated from honey bees. Front Microbiol.

[57] Corby-Harris V, Maes P, Anderson KE (2014). The bacterial communities associated with honey bee (*Apis mellifera*) foragers. PLoS One.

[58] D’Alvise P, Böhme F, Codrea MC (2018). The impact of winter feed type on intestinal microbiota and parasites in honey bees. Apidologie.

[59] Maes PW, Floyd AS, Mott BM, Anderson KE (2021). Overwintering honey bee colonies: Effect of worker age and climate on the hindgut microbiota. Insects.

[60] Rothman JA, Carroll MJ, Meikle WG (2018). Longitudinal effects of supplemental forage on the honey bee (*Apis mellifera*) microbiota and inter- and intra-colony variability. Microb Ecol.

