# Supplementary material for: Geographical and Seasonal Analysis of the Honeybee Microbiome
Source: Microb Ecol. 2022 Mar 14;85(2):765–78. doi: 10.1007/s00248-022-01986-x (PMC9957864; doi:10.1007/s00248-022-01986-x)

Supplementary Information

Supplementary Table 1. Sampling dates for the study

Apiary	Location	T1 inspection	T1 sampling	T2 inspection	T2 sampling	T3 inspection	T3 sampling
A01	North Cork	28/04/19	28/04/19	07/07/19	07/07/19	09/08/19	23/09/19
A02	Galway	02/05/19	02/05/19	22/06/19	22/06/19	24/08/19	24/08/19
A03	Tipperary	04/05/19	28/04/19	06/07/19	29/06/19	07/09/19	01/09/19
A04	Carlow	01/05/19	29/04/19	12/07/19	26/06/19	21/08/19	21/08/19
A05	Dublin	29/04/19	29/04/19	17/06/19	17/06/19	16/08/19	16/08/19
A06	West Cork	19/05/19	19/05/19	20/07/19	20/07/19	12/10/19	12/10/19

Supplementary Fig. 1 Hive metadata from the 2019 survey of 6 hives in southern Ireland. Data are presented as frequency distributions of metadata values as follows: x = value (e.g. FR.bees), y = counts of sampling records with respective x value.

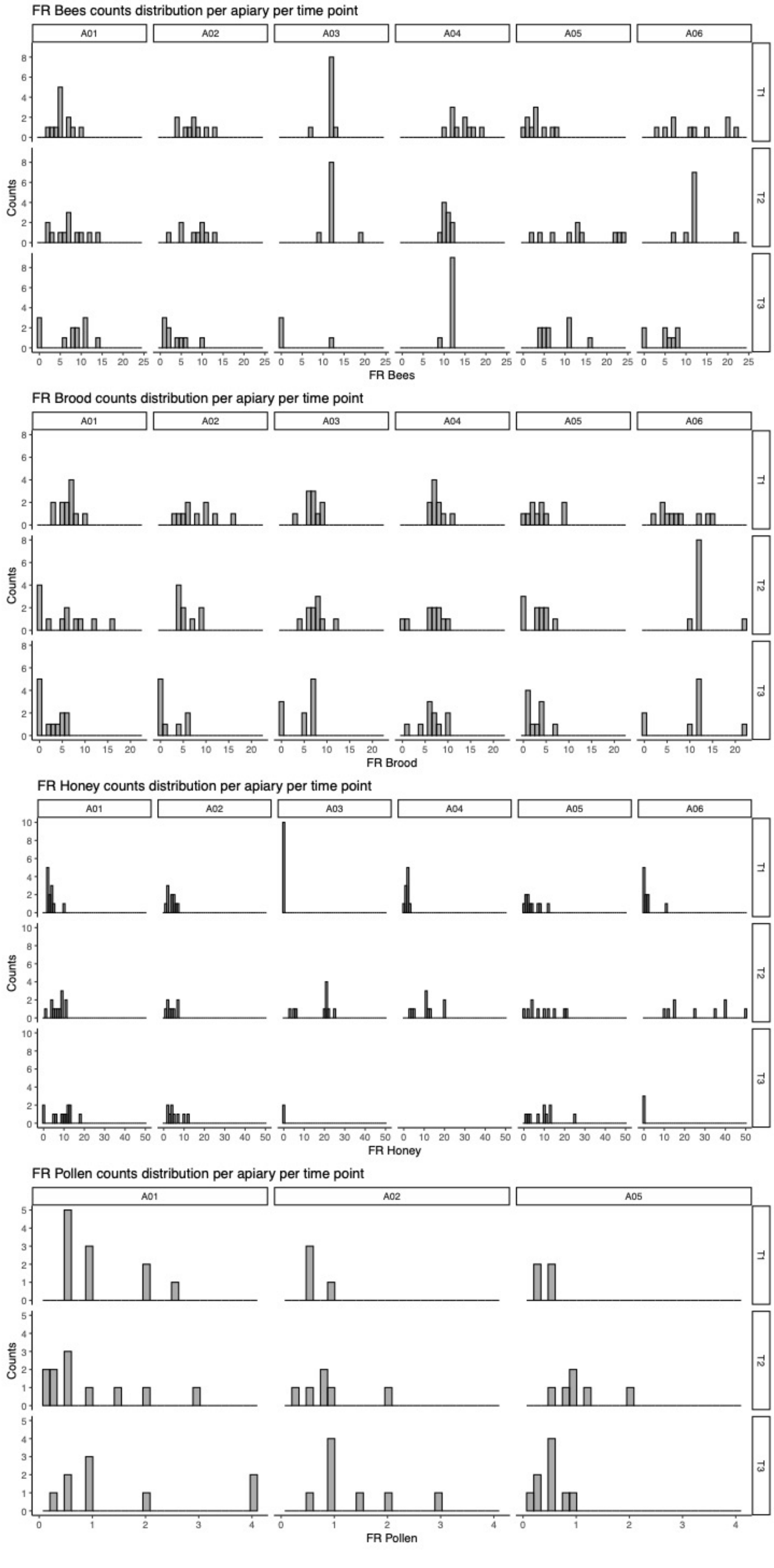

Supplementary Fig. 2 Principal Co-Ordinate Analysis of the honey bee microbiome across time points, separated by apiary.

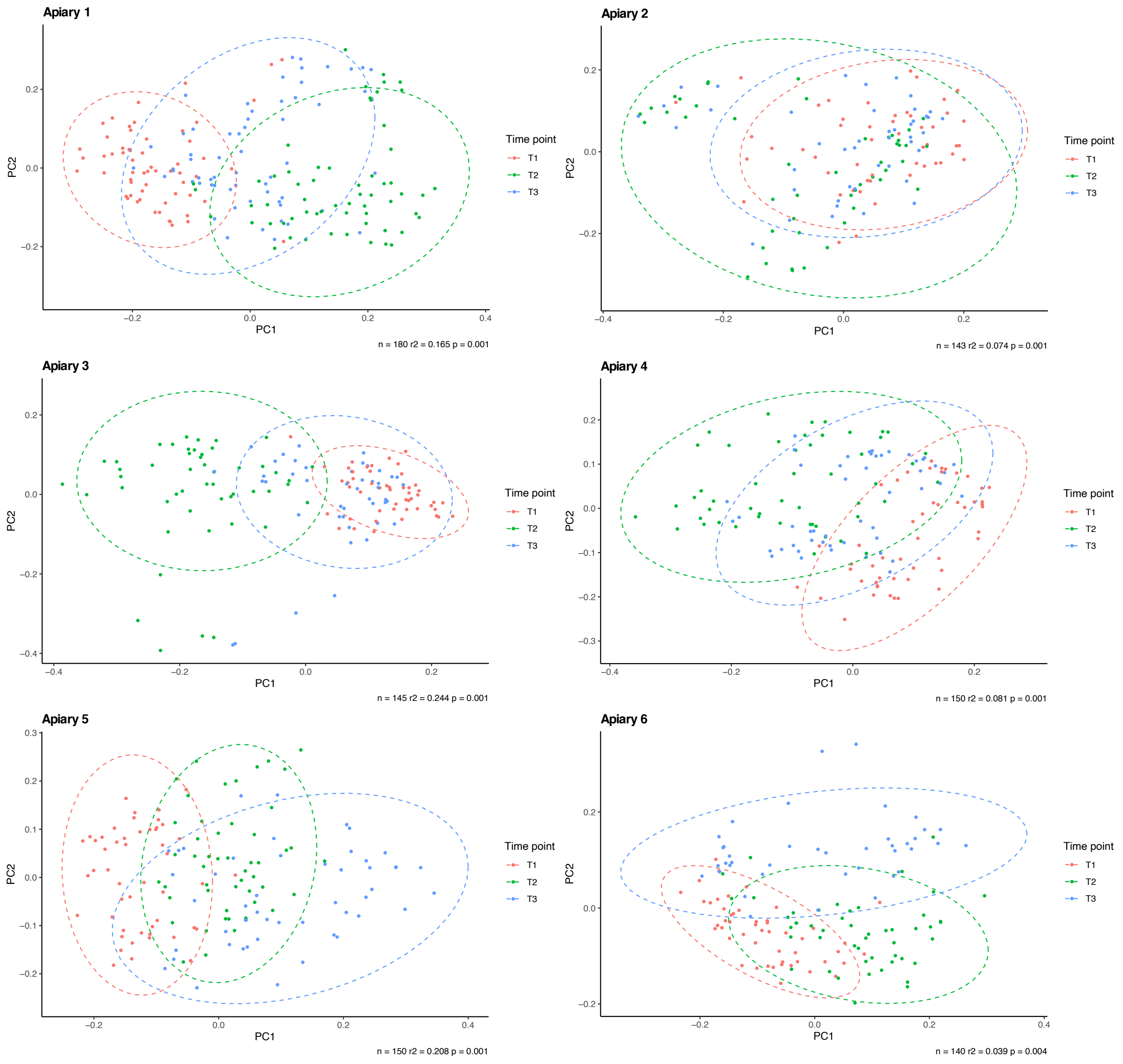

Supplementary Fig. 3 Alpha diversity in whole bee microbiome over time by apiary as measured by A) Simpson Index; B) Chao1 Index. Pair-wise comparisons that reach statistical significance are asterisked; ns = not significant.

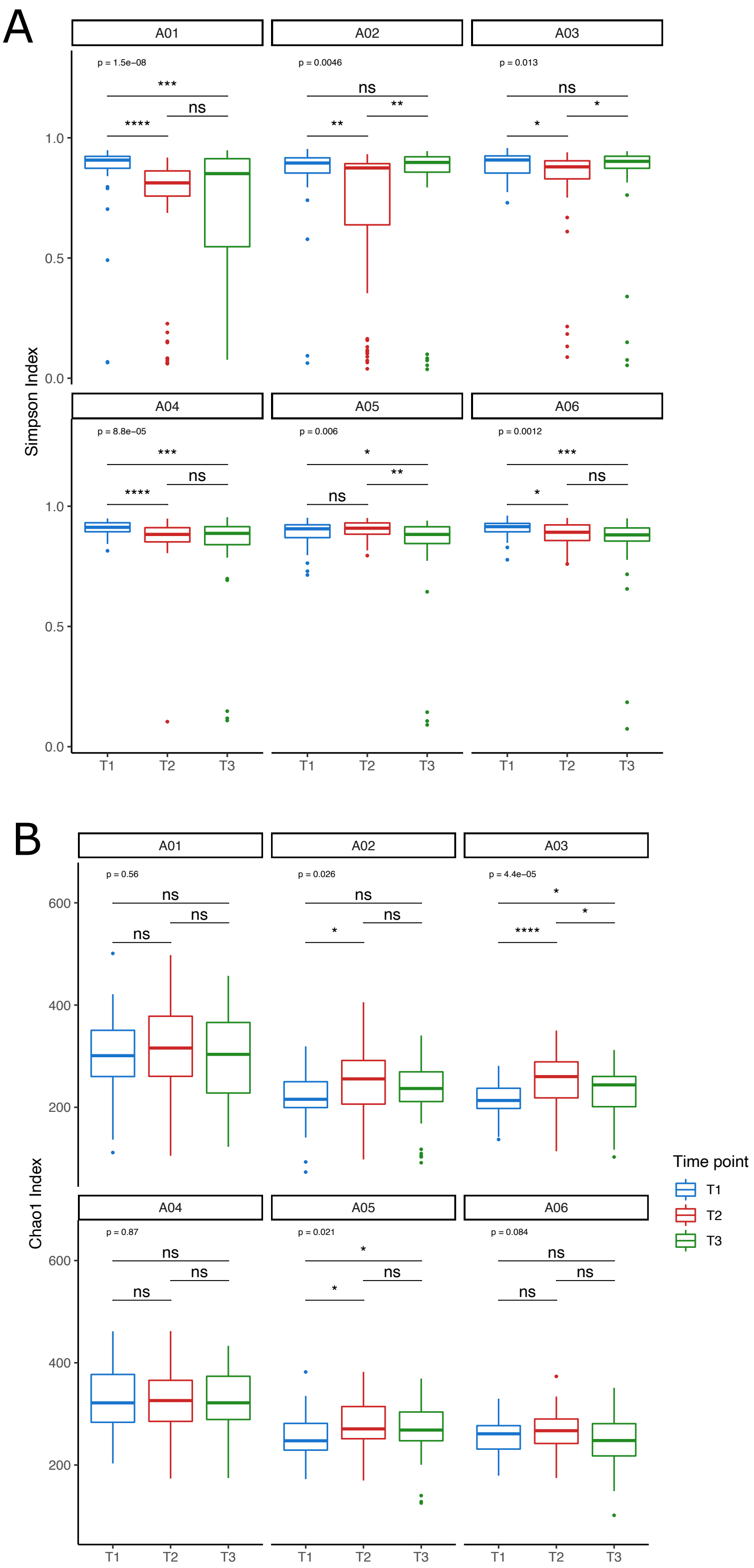

Supplementary Fig. 4 Changes in alpha-diversity (Shannon's Index) over time within hives.

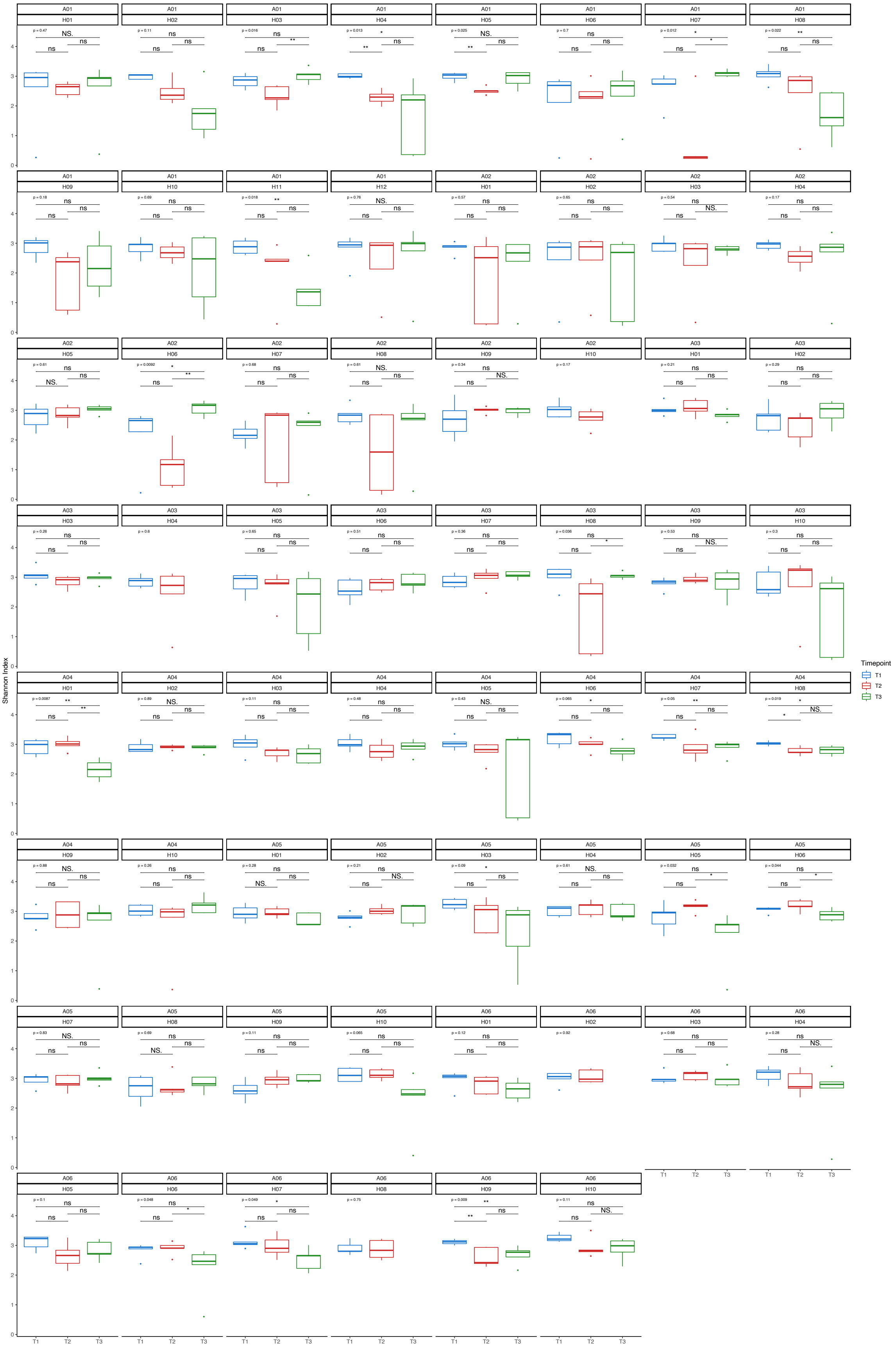

Supplementary Fig. 5 Heat map of Spearman correlations between individual ASVs and hive metadata.

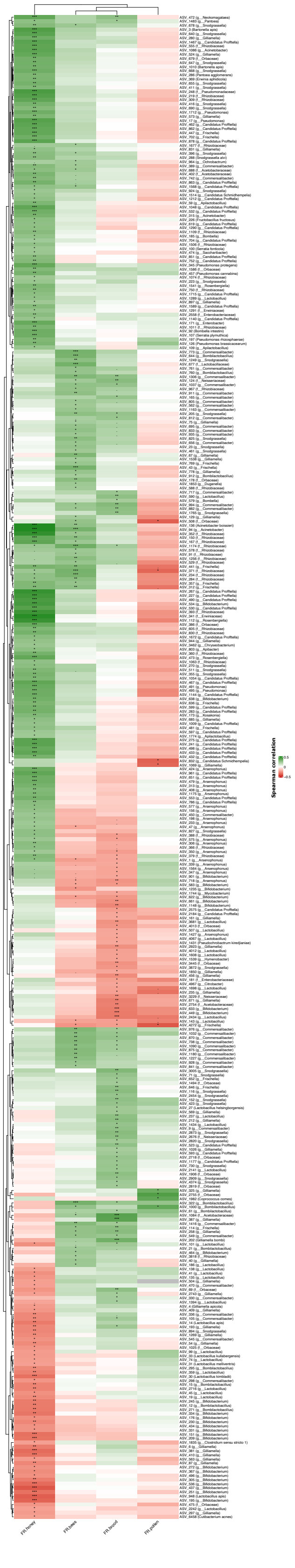

Supplementary Fig. 6 Dot plot representing the mean Spearman correlations (x axis) of ASVs with the same taxonomy classification and their respective frequency (y axis), correlated to the number of frames of A) bees; B) brood; C) honey; D) pollen.

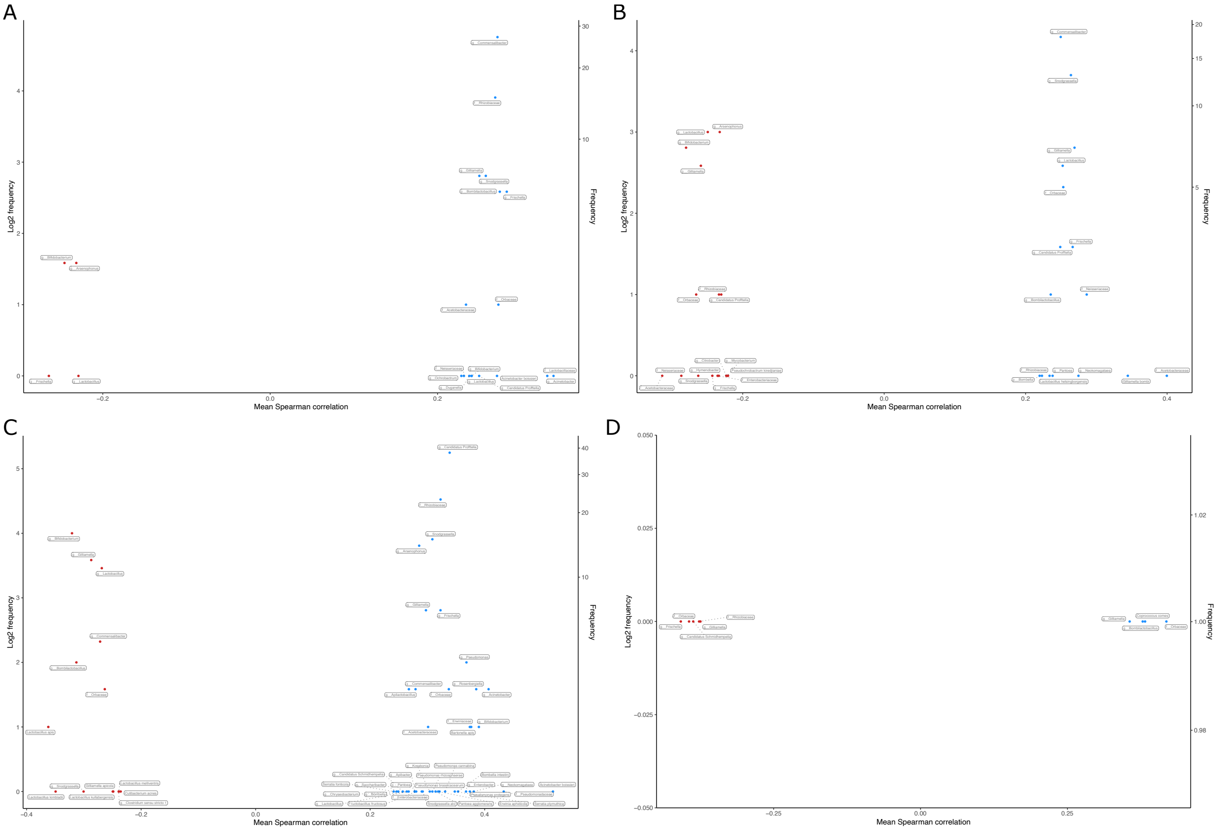

Supplementary Fig. 7 Heat map of Spearman correlations between hive health metadata values in T1, T2 and T3.

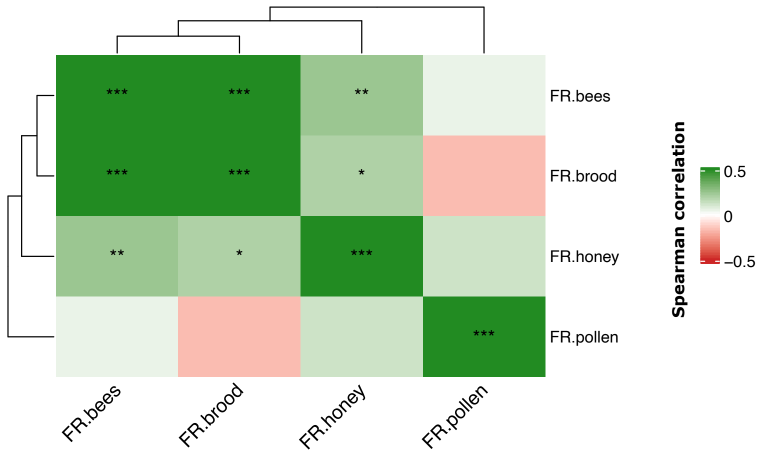

Supplementary Fig. 8 Microbiota composition at Family-level per apiary in WB samples.

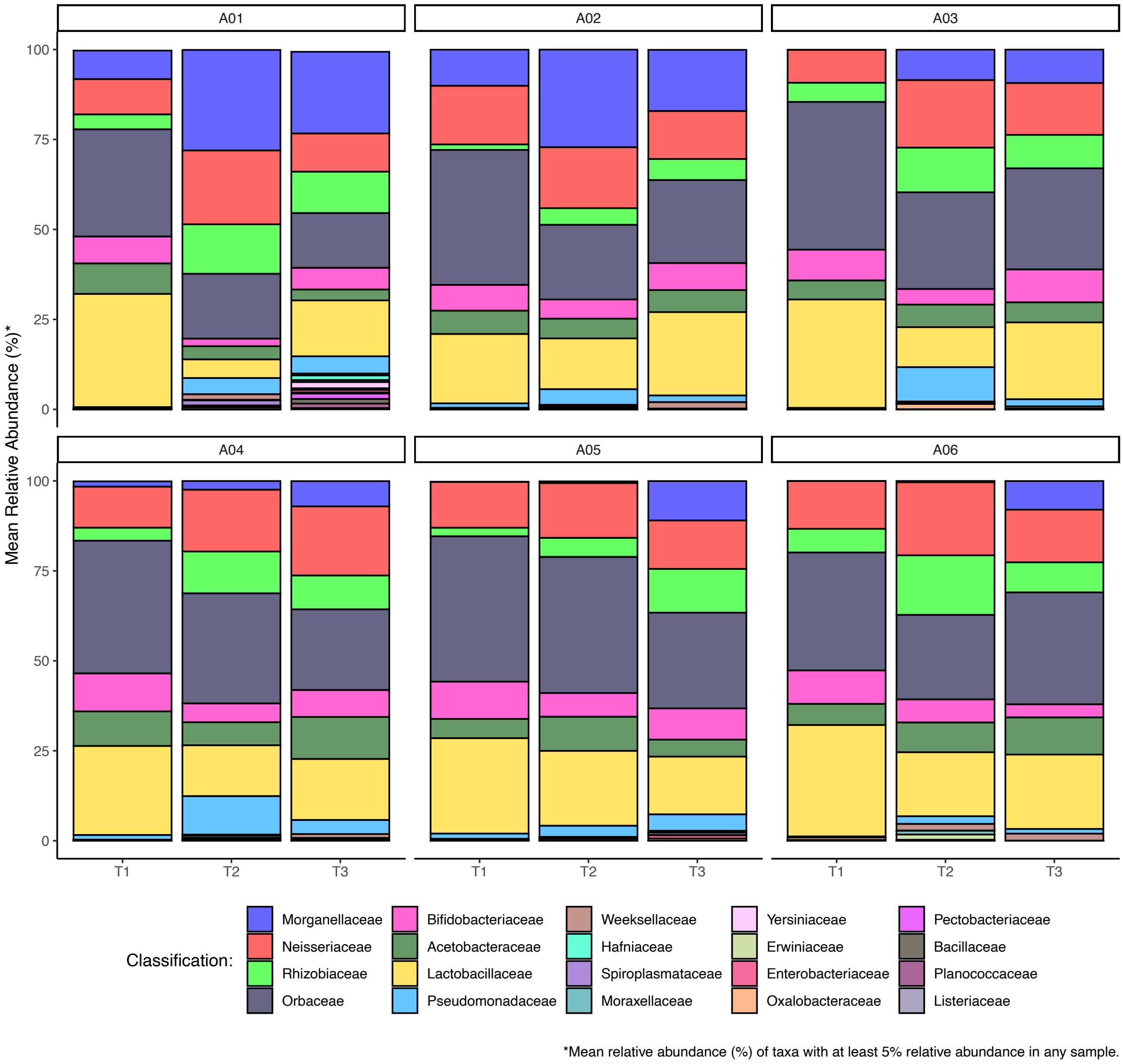

Supplementary Fig. 9 Microbiota composition at Genus-level per apiary in WB samples.

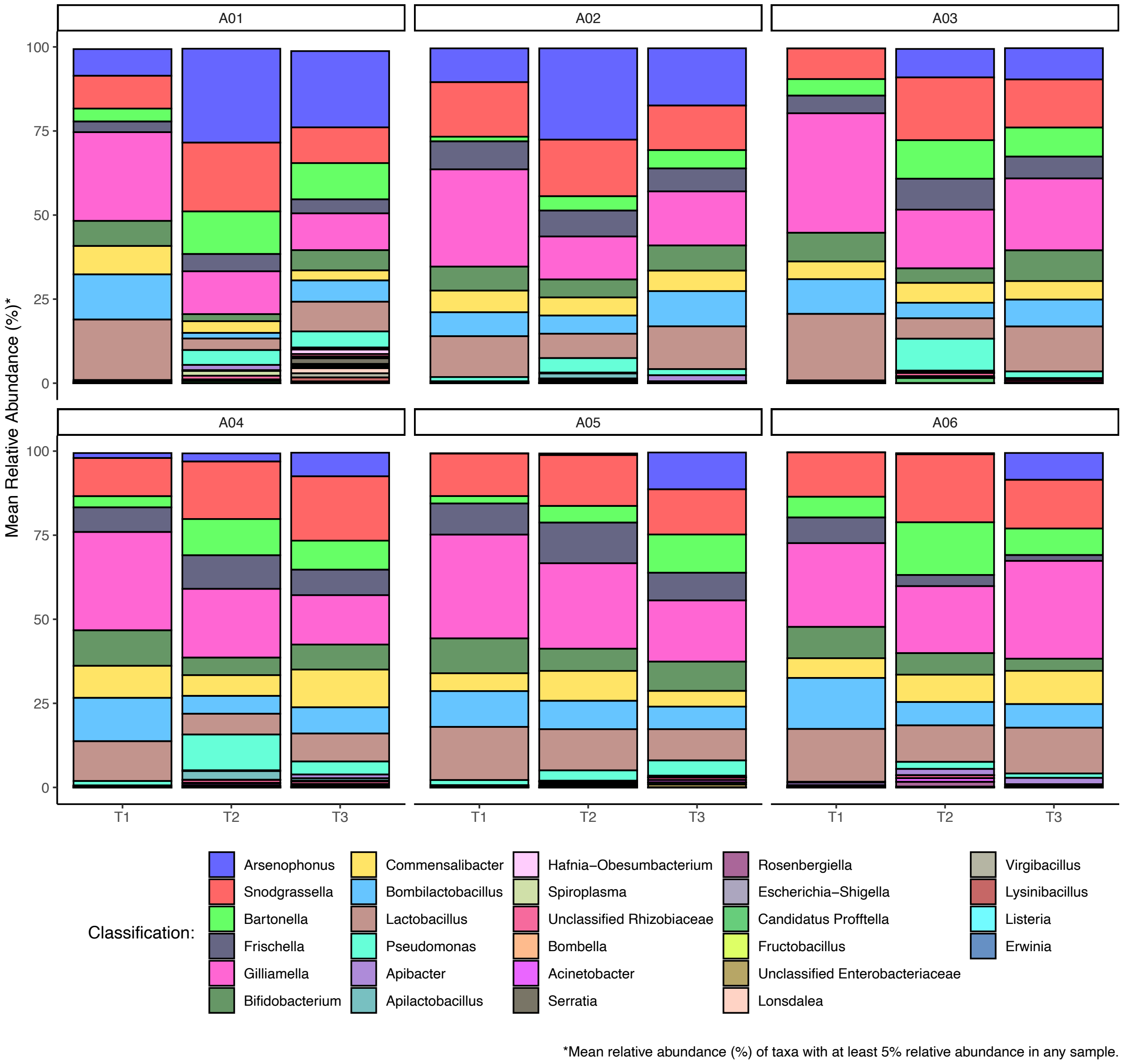

Supplementary Fig. 10 Spearman correlations between agglomerated taxa with at least 1% relative abundance in at least 10% of the samples. *** = p < 0.001; ** = p < 0.01; * = p < 0.5; . = p < 0.1.

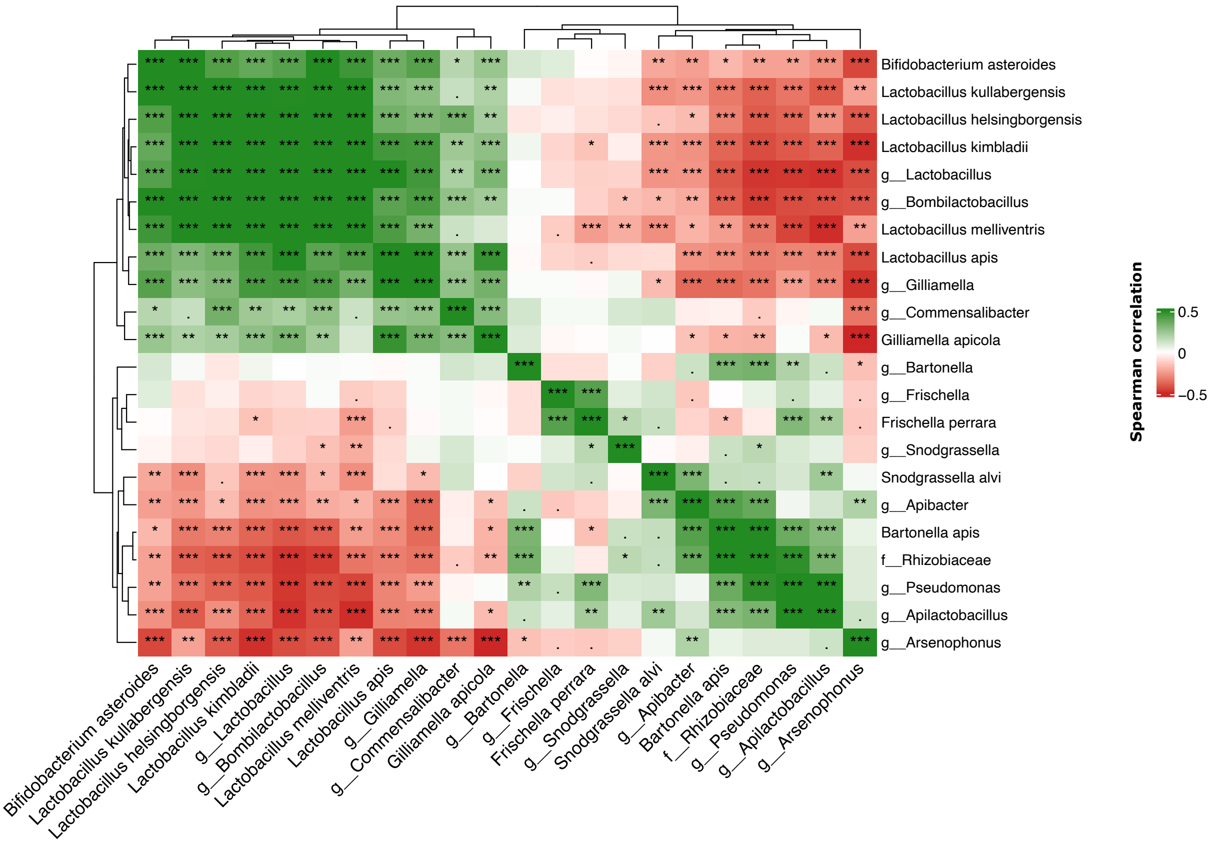

Supplement: Supplementary file 1 — Supplementary file1 (PDF 902 KB) [file 248_2022_1986_MOESM1_ESM.pdf]
